# The application of 18F-FDG PET/CT in ovarian immature teratomas when pathological examination results contradict clinical observations: a case report

**DOI:** 10.1097/MD.0000000000009171

**Published:** 2017-12-15

**Authors:** Manni Wang, Shaoxiong Jiang, Yiwen Zhang, Chong Jiang, Fan Xia, Weiliang Lyu, Xuelei Ma

**Affiliations:** a Cancer Center, West China Hospital, Sichuan University and Collaborative Innovation Center; b Emergency Department; c Department of Nuclear Medicine, West China Hospital, Sichuan University, Chengdu, People's Republic of China.

**Keywords:** lymph metastasis, ovarian immature teratoma, pathology, PET/CT

## Abstract

**Rationale:**

Fluorine-18 fluorodeoxyglucose positron emission tomography/computed tomography (18F-FDG PET/CT) could reveal potential lymph node involvement and assisted locating sample sites for pathological examinations.

**Patient concerns:**

Help choose the right treatment strategies for patients. To better stage immature ovarian teratomas with 18F-FDG PET/CT when lymphatic metastasis is suspected while lymph node biopsy results are negative.

**Diagnoses:**

The ultimate pathological diagnosis was left ovarian cancer, an immature teratoma (IMT) Grade 1.

**Interventions:**

Surgery was the initial treatment option. Chemotherapy (BEP scheme: Bleomycin 30 mg d1, 7 + Etoposide 100mg d1-6 + Cisplatin 50mg d1-3) was then administered.

**Outcomes:**

The post-operational pathological examination additionally showed a small number of tumor cells in para-aortic lymph nodes. The end-of-treatment disclosed no recurrent tumors and serum levels of AFP (2.9 ng/mL), hCG (0.12 mIU/L), and CA-125 (11.4 IU/mL) were normal.

**Lessons:**

18F-FDG PET/CT successfully detected lymphatic metastasis when lymph node biopsy results were negative, which would be of great significance in detecting metastasis and monitoring reoccurrence of ovarian immature teratomas.

## 1 Introduction

The ovarian teratoma is one of the most common germ cell neoplasms worldwide.^[1]^ It contains undifferentiated tissues derived from each of the 3 germ cell layers: ectoderm, mesoderm, and endoderm.^[2]^ Teratomas are classified as mature and immature teratomas according to the differentiation level of their components. Mature teratomas are generally benign, with mature cystic teratomas (also referred to as dermoid cyst) being the most common type of benign ovarian malignancies.^[3]^ The immature teratoma, on the contrary, accounts for <1% of all ovarian cancers^[4]^ and more likely to present malignant behaviors.^[5,6]^ The immature teratoma is often unilateral in occurrence^[7]^ and affects a younger age group, usually during the first 2 decades of life.^[8]^ The most common symptoms noted were abdominal distension and masses (81%).

A noninvasive method is therefore needed to evaluate the primary malignancy and lymphatic metastasis. Fluorine-18 fluorodeoxyglucose positron emission tomography/computed tomography (18F-FDG PET/CT) has the advantage of providing metabolic information of immature teratomas when traditional imaging techniques fail to identify the small foci of fat tissue within the solid calcifications and biopsy results contradict our clinical observations. It is suggested that if the grade advances to the stage beyond Ia, chemotherapy should be recommended,^[9]^ so the precise staging by detecting the lymphatic involvement with the PET/CT-guided biopsy is essential for making treatment strategies and monitoring patients’ prognosis.

In this report, we described a case of ovarian immature teratoma in a 17-year-old female. The application of PET in this clinical setting helped both in presenting metabolic abnormalities and locating potentially problematic lymph nodes. Based on the results we presented in this report, 18F-FDG PET, with the anatomic and functional information it provided, could contribute to the understanding of ovarian immature teratomas and therefore establish clinical decisions.

This case report was approved by institutional ethics committee and no written informed consent was required.

## 2 Case

A 16-year-old girl (G0P0) presented a 5-month history of severe lower abdominal pain, dizziness, and headache in August 2016. There was no associated change in the menstrual cycle, color, quantity, and texture. No significant family history of gynecological cancer or surgical history was present and her overall health was good. Neurological examination showed normal muscles tone, strength, movements, and coordination.

B-ultrasound revealed a giant cystic and solid mass located in the pelvic cavity, with heterogeneous hyperechogenicity. The mass measured 4.1∗3.3∗3.4 cm and no significant ascites was present. Laboratory tests revealed elevated serum levels of α-fetoprotein (AFP) (1329.7 ng/mL; normal <8 ng/mL), carcinoembryonic antigen (8.5 ng/mL; normal <3.4 ng/mL), CA-125 (266.2 IU/mL; normal <35 IU/mL), and CA-19-9 (1329.7 IU/mL; normal <22 IU/mL), but human chorionic gonadotropin (hCG) was within the normal range (2.0 mIU/L).

A FDG-PET scan was accordingly performed which revealed markedly increase of FDG uptake in a solid cystic mass in the abdominopelvic cavity with intralesional fat and high-density component. At the same time, the hypermetabolism of enlarged retroperitoneal lymph nodes of the bilateral pelvic wall and bilateral iliac fossa was observed. The standardized uptake value (SUV) max was 5.8. Whole body 18F-FDG PET/CT indicated enlarged spleen and small amounts of pelvic effusion as well, but did not show pathological FDG uptake in other regions.

Surgery was considered the initial treatment option. The patient then went through a left abdominal adnexectomy, with para-aortic lymph node dissection and pelvic lymph node dissection. During the operation, we observed a 25-cm diameter cyst, with uneven surface and disseminated granule nodules at its root originating from the left ovary. The uterus and right ovary were of normal sizes and peritoneal implants were absent. The intraoperational frozen sections identified only hyperplastic lymphadenopathy without any evidence of metastatic tumor cells. Considering the hypermetabolism of enlarged retroperitoneal lymph nodes of bilateral pelvic wall and bilateral iliac fossa observed in previous 18F-FDG PET/CT, we suspected that the actual condition of patients might be worse than the pathological diagnosis and therefore performed a second pathological examination of the paraffin section at different sites. This time, the postoperational examination additionally showed reactive hyperplasia as well as a small number of tumor cells in para-aortic lymph nodes. The ultimate pathological diagnosis was left ovarian cancer, an immature teratoma (IMT) grade 1. The immunohistochemical staining of tumor tissues showed p63 (+), Ki-67 (+, 3%), CK7 (+), CK (+), CD10 (−), GFAP (+), +Vim (+), S-100 (+), NSE (+), p53 (−), Vim (+), Tg (+), and CA125 (+).

**Figure 1:**
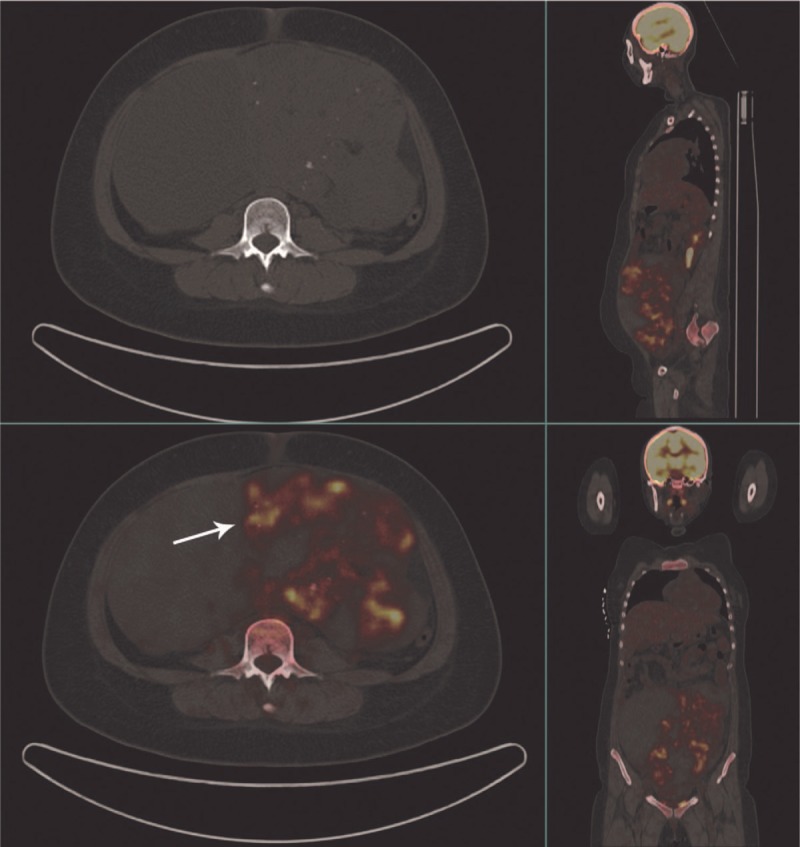
Before surgery, CT images demonstrated a large mass with a maximum diameter of 25 cm with solid, cystic, fat, and calcified components. 18F-FDG PET/CT showed pathological FDG uptake in solid components of the abdominopelvic mass. Intensely increased FDG uptake was also seen in the retroperitoneal lymph nodes of the bilateral pelvic wall and bilateral iliac fossa. CT = computed tomography, 18F-FDG PET/CT = fluorine-18 fluorodeoxyglucose positron emission tomography/computed tomography.

**Figure 2:**
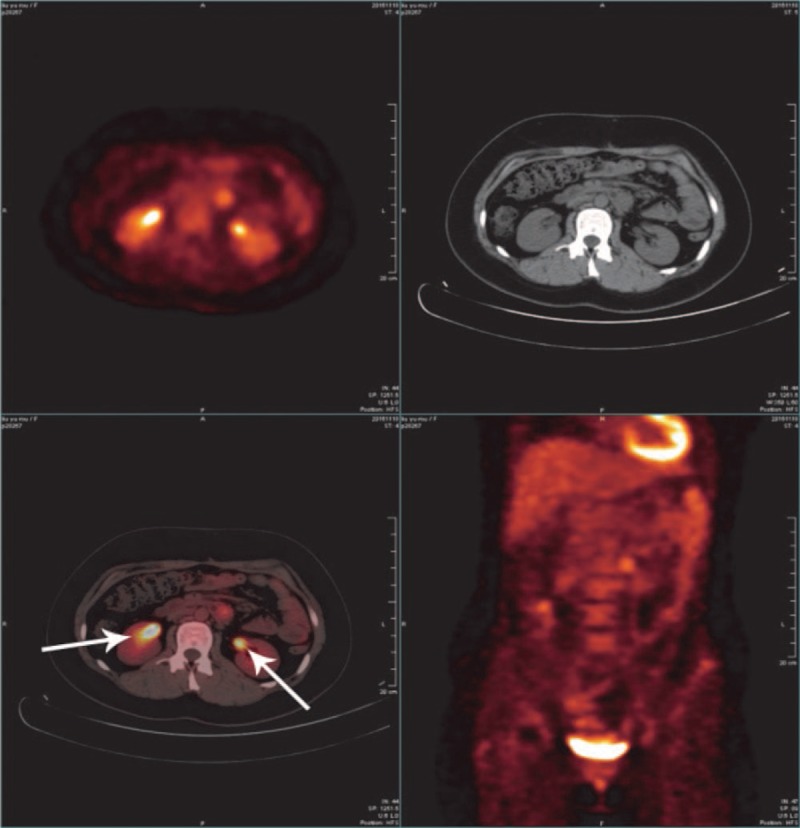
The postoperational 18F-FDG PET/CT presented 2 enlarged para-aortic lymph nodes at the level of L2 and L3 with marked accumulation of FDG and a maximum SUV of 3.5. 18F-FDG PET/CT = fluorine-18 fluorodeoxyglucose positron emission tomography/computed tomography, SUV = standardized uptake value.

The postoperational 18F-FDG PET/CT was performed to determine the presence of metastasis and metabolic activity of the previous mass. Two enlarged left lymph nodes of abdominal aorta at the level of L2 and L3 seen on CT showed accumulation of FDG, with a maximum SUV of 3.5, suggesting potential lymphatic spread. There was no evidence of any pathological FDG uptake in the region of uterus and bilateral uterine appendages and no other evidence of metastasis was found (Figs. 1 and 2).

**Figure 3:**
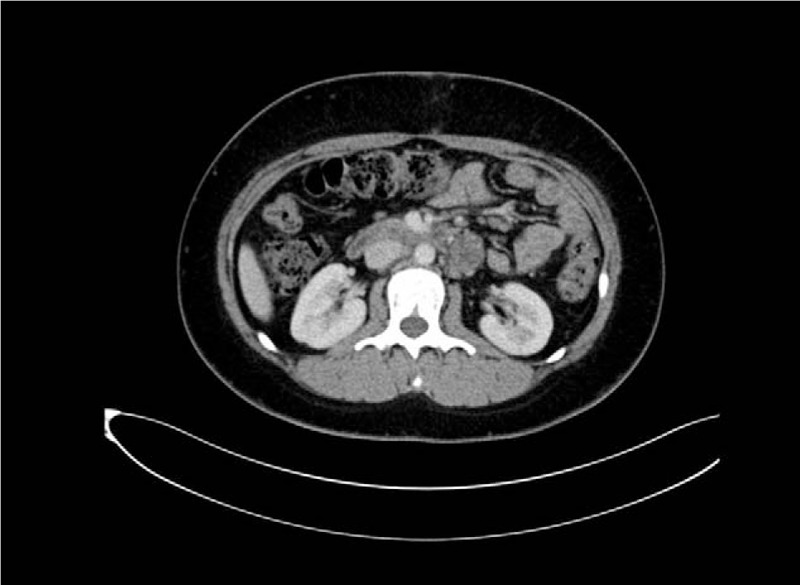
The end-of-treatment follow-up CT scans disclosed no recurrent tumors. CT = computed tomography.

Chemotherapy (BEP scheme: bleomycin 30 mg D1–7 + etoposide 100 mg D1–6 + cisplatin 50 mg D1–3) was then administered. The patient was treated with 3 cycles of BEP. The end-of-treatment follow-up CT scans (Fig. 3) disclosed no recurrent tumors and serum levels of AFP (2.9 ng/mL), hCG (0.12 mIU/L), and CA-125 (11.4 IU/mL) were normal. There was no recurrence after 8 months of follow-up.

## 3 Discussion

Immature teratomas differ from mature teratomas in that they only account for <1% of ovarian terotomas, more likely to demonstrate malignant behavior in clinical observations, affect a younger age group, and contain immature or embryonic tissues in histological examinations.^[1]^ The prognosis of patients with immature teratomas depends on the stage and grade.^[10–12]^ Simple resections are considered the most effective treatment for mature teratomas, while immature teratomas might require a comprehensive combination of surgery, chemotherapy, and radiotherapy in order to reach optimal results.^[13]^ Much more desirable prognostic results of surgery alone, without chemotherapy, have been reported in patients with early-stage IT than grades 1 and 2.^[14–16]^ The 5-year survival rate of immature teratoma stage I is 90% to 95%, whereas advanced-stage survival drops to about 50% in grade 1^[17]^ as is in this case. Norris et al^[10]^ reported that the rate of recurrence of immature teratoma was 18% in grade 1. As a result, it is suggested that if the grade advances to the stage beyond Ia, chemotherapy should be recommended,^[9]^ so in this case, chemotherapy (BEP scheme: bleomycin 30 mg d1,7 + etoposide 100 mg D1–6 + cisplatin 50 mg D1–3) was then given to the patient. On this sense, the early diagnosis of immature teratomas by using existing imaging techniques is essential for making treatment strategies and prolonging patients’ survival.

At CT and MRI, immature teratomas possess characteristic appearance. A large, irregular solid mass containing coarse calcifications and small foci of fat could be observed.^[18–21]^ Immature teratomas are typically large (14–25 cm) in size and have prominent solid components with cystic elements^[22]^ and the tumors frequently present perforation of the capsule, which makes their borders poorly defined.^[23]^ The immature teratoma is usually filled with lipid constituents and therefore demonstrates fat density at CT and MRI. These lipid materials include sebaceous components inside the cyst cavity, adipose tissues within the cyst wall, or dermoid plugs.

Contrast-enhanced CT is commonly used in the evaluation and follow-up of teratoma patients. However, small foci of fat within the solid calcifications may be difficult to recognize. In some previously reported cases, the preoperational CT scan and MRI failed to indicate the malignancy.^[24]^ On the other hand, although biopsy remains the diagnostic gold standard, it has some limitations such as the potential injury to surrounding tissues, nerves, and blood vessels when performing before surgery, and the failure to take samples at proper locations as stated in this case. The clinical diagnosis could be made, based on the combination of traditional imaging techniques with the metabolic activities of masses on FDG PET/CT, confirming the nature of foci and therefore improving clinical decision of teratomas.

The combination of PET and CT imaging has the advantage of providing more precise morphologic and functional details of lesions and their surrounding tissues. PET scanning with 18F-FDG has become an effective and well-targeted imaging modality in many adult cancer patients and has an increasing application in pediatric patients with solid tumors.^[25,26]^ A recent study showed that 18F-FDG PET/CT could effectively help present malignant involvement of the peripheral nerves when results of MRI or CT are negative.^[27]^ Previous reports have demonstrated the effective application of PET in adults with germ-cell tumors, rhabdomyosarcoma, adenocarcinomas, and other neoplasms resulting from somatic malignant transformation in teratomas.^[25]^ Jiménez-Bonilla et al^[28]^ discovered that PET/CT identified recurrence in 44.3% of scans performed without prior clinical suspicion and ruled out recurrence in 24.2% of scans performed with prior clinical suspicion. Other prominent advantages of PET include its application in the differentiation between benign and malignant diseases, initial staging, therapeutic monitoring, detection of residual neoplasm, and surveillance for recurrence and metastasis.^[29]^

In this case, FDG PET confirmed very high metabolic activity of retroperitoneal lymph nodes of bilateral pelvic wall and bilateral iliac fossa, which led to our suspicion on the first pathological examination results suggesting only reactive hyperplasia of lymph nodes without any evidence of tumor cells. We suspected that the actual condition of patients might be worse than the pathological diagnosis and the postoperational examination of the paraffin section was accordingly performed. This second pathological examination of lymph nodes at different sites confirmed metastasis in para-aortic lymph nodes, which led to the ultimate diagnosis of left ovarian cancer, an IMT grade 1.

The reason for the contradiction is probably that, although the histopathological examination of the lymph node specimens provides basic information of diagnosis and staging, it sometimes fails to accurately locate abnormal lymph nodes that contain tumor cells. Owing to the fact that, at relatively early stages, the focal lymphatic metastasis occurs only with a small number of tumor cells, the conventional method of obtaining a few lymph node sections for pathological examination sometimes misses out certain lymph nodes that contain tumor-related components. Since taking serial sections of regional lymph nodes is generally impractical, a much precise and well-targeted method should be added to help locate potentially problematic lymph nodes. In this case, we utilized 18F-FDG PET/CT to predict the eventual diagnosis and to clarify recurrence when it was highly suspected or our clinical observation contradicted histological findings. In addition, nodal status and resemblance of metastatic lymphadenopathy to inflammatory abnormalities pose difficulty in nodal staging which is directly associated with distant recurrence and overall survival and largely impacts the selection of surgical options and following treatments.

In conclusion, molecular imaging with 18F-FDG PET/CT is considered effective and sometimes better-directed in staging immature teratomas when lymphatic metastasis is suspected but lymph node biopsy results are negative. In this way, metabolic information provided by 18F-FDG PET/CT assists the selection of the most appropriate treatment. Meanwhile, considering its role in detecting reoccurrence, 18F-FDG PET/CT is also suggested to be added into personalized follow-up surveillance to improve patients’ overall survival.
